# Associations Between Daily Interpersonal Stressor Control and Memory Lapses in Daily Life

**DOI:** 10.1093/geroni/igaf122.183

**Published:** 2025-12-31

**Authors:** Eric Cerino, Faith Shannon, Gillian Porter, Dakota Witzel, Robert Stawski, Jonathan Rush, Jacqueline Mogle, David Almeida

**Affiliations:** Northern Arizona University, Flagstaff, Arizona, United States; Northern Arizona University, Flagstaff, Arizona, United States; Northern Arizona University, Flagstaff, Arizona, United States; South Dakota State University, Brookings, South Dakota, United States; Utah State University, Logan, Utah, United States; University of Victoria, Victoria, British Columbia, Canada; RTI Health Solutions, Durham, North Carolina, United States; The Pennsylvania State University, University Park, Pennsylvania, United States

## Abstract

Studying modifiable psychosocial correlates of cognitive health in daily life is crucial to inform targets for dementia prevention. Perceived control is an important psychosocial resource for cognitive health. Prior work, however, focuses largely on global aspects of control, ignoring influences of more dynamic aspects of control associated with specific experiences in daily life (i.e., daily stressor control). We used data from the National Study of Daily Experiences (N = 1,236, Mage=67.54, SE = 10.34, 57.38% Female) to address this gap and examine daily associations among stressor control and memory lapses. Over eight consecutive evenings, respondents reported perceived control over interpersonal stressors they had experienced and two types of memory lapses (retrospective, prospective) as well as the impact of these lapses each day (irritation, interference). We used multilevel modeling with statistical adjustment for number of stressors, gender, education, race, and global control. On average, people with higher stressor control reported significantly lower irritation ratings from retrospective memory lapses (Est.=-0.47, SE = 0.15, p<.01). Within-person stressor control and age significantly interacted (Est.=-0.03, SE = 0.01, p=.01) such that days when stressor control was higher than usual were associated with less irritation from prospective memory lapses only among comparatively older adults (Est.=-0.66, SE = 0.31, p<.05). Results indicate that daily stressor control may serve as a psychosocial resource related to less irritation when forgetting past information and future intentions. We discuss the value of promoting perceived control over daily interpersonal stressors to protect against detrimental impacts of memory lapses and encourage healthy cognitive aging in daily life.

